# Insights on therapeutic potential of clemastine in neurological disorders

**DOI:** 10.3389/fnmol.2023.1279985

**Published:** 2023-09-28

**Authors:** Sufang Jiang, Xueji Wang, Tianyu Cao, Rongtian Kang, Lining Huang

**Affiliations:** ^1^Department of Anesthesiology, The Second Hospital of Hebei Medical University, Shijiazhuang, Hebei, China; ^2^The Key Laboratory of Neurology, Ministry of Education, Shijiazhuang, Hebei, China

**Keywords:** clemastine, central nervous system diseases, neuroprotective, myelination, cognition

## Abstract

Clemastine, a Food and Drug Administration (FDA)-approved compound, is recognized as a first-generation, widely available antihistamine that reduces histamine-induced symptoms. Evidence has confirmed that clemastine can transport across the blood–brain barrier and act on specific neurons and neuroglia to exert its protective effect. In this review, we summarize the beneficial effects of clemastine in various central nervous system (CNS) disorders, including neurodegenerative disease, neurodevelopmental deficits, brain injury, and psychiatric disorders. Additionally, we highlight key cellular links between clemastine and different CNS cells, in particular in oligodendrocyte progenitor cells (OPCs), oligodendrocytes (OLs), microglia, and neurons.

## 1. Introduction

**Figure 1:**
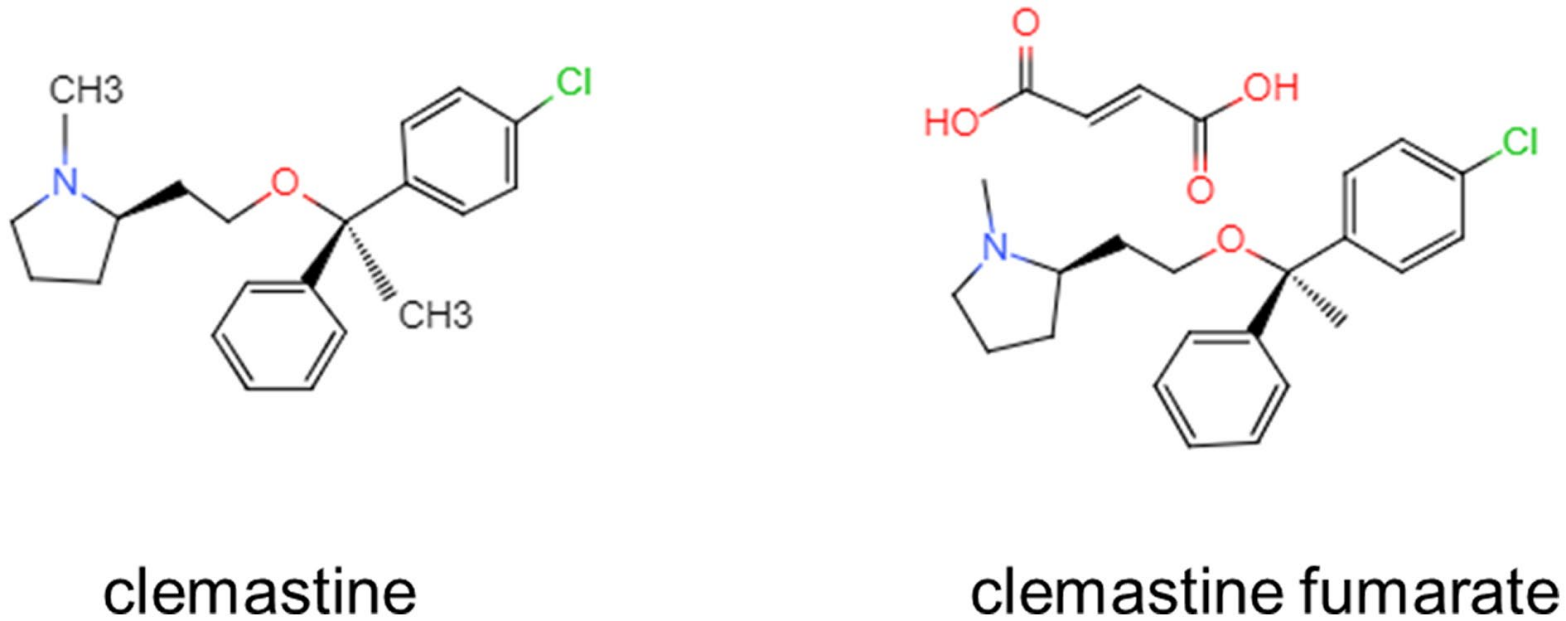
The chemical structures of clemastine and clemastine fumarate.

Clemastine, known as 2-[2-[1-(4-chlorophenyl)-1-phenylethoxy]ethyl]-1 -methylpyrrolidine, is a Food and Drug Administration (FDA)-approved compound. It is a first-generation, widely available histamine H1 receptor antagonist that prevents the symptoms caused by high histamine levels (Simons, 2004). Clemastine is often produced as clemastine fumarate to improve its solubility and bioavailability. The molecular structure of clemastine and clemastine fumarate are shown in Figure 1. Although the initial research on clemastine was primary mainly focused on its antihistamine properties and side effects, following a high-throughput screen it has been identified, as a potential treatment for multiple sclerosis (MS) or more specifically as a drug promoting remyelination (Mei et al., 2014), in an attempt aimed at repurposing known molecules. Then various preclinical and clinical researches have investigated the therapeutic potential of clemastine in neurological disorders, including neurodegenerative disease, neurodevelopmental deficits, brain injury, and psychiatric disorders (Mei et al., 2016; Cree et al., 2018; Pan et al., 2020; Shimizu et al., 2020; Chen J. F. et al., 2021; Chen L. et al., 2021; Leng and Edison, 2021; Wu et al., 2021b; Hong et al., 2022; Bohlen et al., 2023). However, a full appreciation of therapeutic potential of clemastine in neurological disorders needs to reveal the underlying mechanisms.

Oligodendrocytes (OLs) are the myelin-forming glial cells of the central nervous system (CNS), which derive from the differentiation of oligodendrocyte progenitor cells (OPCs) throughout the lifespan (Young et al., 2013; Cristobal and Lee, 2022). The balance of myelination, demyelination, and remyelination is often regulated by cell–cell interactions with other glia (Molina-Gonzalez et al., 2022). Microglia are known to undergo significant transcriptional changes during demyelination and remyelination. On one hand microglia facilitate developmental myelination and remyelination through engulfing excessive unneeded myelin and myelin debris (Xu et al., 2022). However, in pathological conditions of neurological disorders, microglia can be activated and produce pro-inflammatory cytokines, which impair myelination and result in progressive demyelination (Kent and Miron, 2023). In this review, we present preclinical and clinical evidence to illustrate the beneficial effects of clemastine in neurological disorders. We highlight that clemastine’s actions are mainly associated with promoting oligodendrocyte differentiation and maturation, inhibiting microglia induced neuroinflammation, and decreasing the apoptosis of neurons. Additionally, we summarize related molecular mechanisms underlying therapeutic potential of clemastine in animal models of neurological disorders (Table 1) and cell experiments (Table 2).

**Table 1:** Clemastine in central nervous system disorders.

Neurological disorder	Animal model	Doses and frequency	Major findings	Mechanisms	Ref.
Multiple sclerosis	*EAE mouse model*	10 mg/kg/d, oral, daily, for 32 days	Prevented axonal loss, accelerated remyelination, attenuated EAE clinical scores	CHRM1 inhibition	Mei et al. (2016)
	*Lysolecithin-induced demyelination mice*	10 mg/kg/d, gastric gavage, daily, for 14 days	Increase mature oligodendrocytes, accelerated remyelination, increased axon preservation	–	Jensen et al. (2018)
Amyotrophic lateral sclerosis	SOD1^G93A^ mouse model	10 mg/kg/d, intraperitoneally injection, five times a week, from 40 days of age until end stage of the disease	Reduced microgliosis, modulates microglia-related inflammatory genes, enhances motor neuron survival	–	Apolloni et al. (2016b)
	SOD1^G93A^ mouse model	50 mg/kg/d, intraperitoneally injection, five times a week, for 80 days or 125 days	Short term treatment decreased inflammation and SOD1 protein, enhanced autophagy, prolonged survival; long treatment produced opposite effects	mTOR signaling pathway	Apolloni et al. (2016a)
*Aged*	*12-month-old mice*	10 mg/kg/d, oral, daily, for 4 months	Enhanced myelination, preserved spatial memory capacity	CHRM1 inhibition	Wang et al. (2020)
Alzheimer's disease	APP/ PS1 mice	10 mg/kg/d, oral, daily, for 4 months	Decrease amyloid-β generation and accumulation, mitigated neuroinflammation, enhanced autophagy, rescued cognitive deficits	Reduced BACE1; Enhanced mTOR-mediated autophagy	Li et al. (2021)
	APP/ PS1 mice	10 mg/kg/d, oral, daily, for 2 months	Ameliorates accumulation of amyloid-β, prevented OPCs senescence, facilitated formation of myelin, rescued cognitive deficits	–	Xie et al. (2021)
	APP/ PS1 mice	10 mg/kg/d, oral, daily, for 3 months	Promoted new myelin formation, rescued cognitive decline	CHRM1 inhibition	Chen J. F. et al. (2021)
Perioperative neurocognitive disorders	Isoflurane anesthesia plus exploratory laparotomy induced mice	10 mg/kg/d, intraperitoneally injection, daily, for 2 weeks	Decreased neuroinflammation, promoted remyelination, improved survival of hippocampal neurons, ameliorated cognitive deficit	Blocked activation of WNT/β-catenin signaling pathway	Wu et al. (2021b)
Essential omega-3 fatty acids (n-3 PUFAs) deficiency related disorder	Dietary n-3 polyunsaturated fatty acids deficient mice	10 mg/kg/d, intraperitoneally injection, daily, for 7 days	Promoted oligodendrocytes maturation, rescued memory deficits in n-3 PUFA deficient animals	–	Madore et al. (2020)
Williams syndrome	Forebrain excitatory neuron-specific *Gtf2i* knockout mice	10 mg/kg/d, oral, daily, for 14 days	Restored myelination properties, increased axonal conductivity, rescued the behavioral deficits	–	Barak et al. (2019)
Autism spectrum disorder	Pitt-Hopkins syndrome mouse model	10 mg/kg/d, intraperitoneally injection, every 24 h for 14 consecutive days	Enhanced differentiation of OPCs, increased myelination activity, rescued electrophysiological deficits	–	Bohlen et al. (2023)
Inhaled anesthetics induced development disorder	Isoflurane induced developmental neurotoxicity in mie	10 mg/kg/d, oral, daily, for 14 days	Reverse cognitive dysfunction induced by isoflurane	Decreased activity of the mTOR signaling pathway	Li et al. (2019)
	Repeated sevoflurane exposure in neonatal mice	10 mg/kg/d, intraperitoneally injection, daily, for 26 days	Enhanced OLs maturation and myelination, alleviated cognitive impairment induced by sevoflurane	–	Zhang et al. (2022)
Normal brain development	C57BL/6 mice	50 mg/kg/d, intraperitoneally injection, daily, for 16 day	Increased oligodendrocyte differentiation, activated microglia	–	Palma et al. (2022)
Major depressive disorder	Socially isolated mice	10 mg/kg/d, oral, daily, for 2 weeks	Induced OPCs differentiation, enhanced myelination, reversed social avoidance behavior	Enhanced the activity of H3K9 histone methyltransferases	Liu et al. (2015)
	Socially defeated mice	10 mg/kg/d, oral, daily, for 5 days	Increased oligodendrogenesis, rescued the behavioral abnormalities	–	Shimizu et al. (2020)
	Chronic unpredictable mild stress induced mice	10 mg/kg/d, intraperitoneally injection, daily, for 5 days	Decreased pro-inflammatory cytokines, suppressed microglial M1-like activation	–	Su et al. (2018)
Schizophrenia	Cuprizone-induced mouse model of schizophrenia	10 mg/kg/d, oral, daily, for 5 days	Increased mature oligodendrocytes, rescued the schizophrenia-like behavioral changes	–	Li et al. (2015)
Hypoxic brain injury	Neonatal hypoxic mice injury model	10 mg/kg/d, oral, daily, for 7 days	Promoted OPCs differentiation, myelination, improved functional recovery	CHRM1 inhibition	Cree et al. (2018)
	Neonatal hypoxic mice injury model	10 mg/kg/d, oral, daily, for 8 days	Enhanced oligodendroglial differentiation, improved functional recovery	CHRM1 inhibition	Wang et al. (2018)
	Adult hypoxia exposure mouse	10 mg/kg/d, oral, daily, for 4 weeks	Increased the newly-formed myelin, prevented motor coordination deficits	–	Chen L. et al. (2021)
Hypoxic-ischemic brain injury	Bilateral common carotid artery ligation rat	1 mg/kg/d, intraperitoneally injection, daily, for 14 days	Reverse hypomyelination, restrain the upregulation of IL-1β	–	Xie et al. (2020)
Spinal cord injury	Spinal cord injury model of rat	10 mg/kg/d, oral, daily, for 28 days	Enhanced myelination, delayed axonal loss, improved functional recovery		Du et al. (2022)
	Spinal cord injury model of rat	10 mg/kg/d, oral, daily, for 7 or 14 days	Enhanced oligodendrocyte differentiation and myelin wrapping	Inhibited CHRM1 and then activated ERK1/2 pathway	Tong et al. (2022)
Remote fear memory	C57BL/6J mice	10 mg/kg/d, intraperitoneally injection	Induced new myelin formation, improved remote memory recall	–	Pan et al. (2020)
Remote memory consolidation	TrkB-deleted mice	10 mg/kg/d, intraperitoneally injection, daily, for 17 days	Promoted maturation of OPCs, improved remote memory recall	–	Hong et al. (2022)
Compression neuropathy	Mouse model of compression neuropathy	10 mg/kg/d, intraperitoneally injection, daily, for 2 weeks	Promoted myelin repair, improved electrophysiologic and histomorphometric changes	–	Lee et al. (2021)
Chemotherapy-induced cognitive impairment	Chemotherapy-induced cognitive impairment of mie	10 mg/kg/d, oral, daily, for 2 weeks	Enhanced oligodendrocyte differentiation, promoted remyelination, rescued cognitive function damage		Chen et al. (2022)
Intracerebral hemorrhage	Intracerebral hemorrhage mice	30 mg/kg/d, intraperitoneally injection, one time	Inhibited microglia-induced inflammatory and apoptosis, enhanced restoration of neuronal function	–	Zhi et al. (2021)

**Table 2:** Clemastine in vitro study.

Cells	Cell origin	Intervention	Clemastine doses and incubation time	Major findings	Mechanisms	Ref.
OPCs	Cerebral cortices from 2-day-old postnatal rat	Co-culture with human neural progenitor cells,	1 μM for 48 hr	Enhanced OPCs differentiation	–	Nicaise et al. (2017)
	Cerebral hemispheres from 1-day-old postnatal SD rats	IL-1β	20 ng/ml for 7 days	Promoted the maturation of primary OPCs	Activated ERK phosphorylation	Xie et al. (2020)
Primary microglial cells	Brain cortex from 1-day old postnatal SOD1^G93A^ mice	P2X7 receptor agonist BzATP	30 μM for 18 hr	Inhibited M1 phenotype, promoted M2 phenotype	–	Apolloni et al. (2016b)
	Cerebral hemispheres from 1-day-old postnatal SD rats	Oxygen glucose deprivation	20 ng/ml for 1 hr	Inhibited activation of microglial cells	Inhibited p38 phosphorylation	Xie et al. (2020)
	Spinal cord from 120-day-old postnatal SOD1G93A mice	–	30 μM for 6 hr	Decreased inflammation and SOD1, enhanced autophagic activity	Inhibited mTOR signaling pathway	Apolloni et al. (2016a)
BV2 microglial cells		Lysed murine RBC	20 or40 μM for 72 hr	Suppressed microglia activation	–	Zhi et al. (2021)
Primary cortical neurons	Brain cortex from 14-days-old postnatal mouse	Co-culture with activated microglia	20 or 40 μM for 72 hr	Protected against neuronal apoptosis induced by activated microglia	–	Zhi et al. (2021)

## 2. Effects of clemastine in experimental studies of neurodegenerative diseases

Ageing is the primary risk factor for a majority of neurodegenerative diseases. Unfortunately, effective strategies for ageing-related neurodegenerative diseases are limited. Recent evidence has indicated that clemastine showed beneficial effects in neurodegenerative diseases by promoting myelination, enhancing autophagy, and mitigating neuroinflammation.

### 2.1. Multiple sclerosis

Multiple sclerosis (MS) is a chronic autoimmune disease characterized by chronic demyelination. The disease pathogenesis involves in oligodendrocyte cell death, myelin sheath destruction, and axonal injury (Jäkel et al., 2019). Almost three million people are estimated to suffer from MS worldwide (Walton et al., 2020). A high-throughput *in vitro* screening system has identified clemastine as a promising candidate for MS therapy (Mei et al., 2014).

Anterior visual pathway is demyelinated in almost all MS patients (Toosy et al., 2014), and visual evoked potentials has been identified as a preclinical, quantitative biomarker for remyelination efficacy in MS patients (Cordano et al., 2022). A clinical phase II, 150 days, double-blind, randomized, placebo-controlled, crossover trial (ReBUILD) in patients with relapsing MS has been completed (NCT02040298). In this trail, patients received either clemastine (5.36 mg orally twice daily) for 90 days followed by a placebo for 60 days, or vice versa. Clemastine treatment reduced the visual-evoked potentials latency delay by 1.7 ms/eye when analyzing the trial as a crossover (Green et al., 2017). Recently the group of the ReBUILD trial (NCT02040298) further verified the therapeutic effects of clemastine in MS patients. Neurofilament light chain (NfL), a marker of neuroaxonal injury, is elevated in patients with MS (Cantó et al., 2019; Kuhle et al., 2019, 2020). Clemastine was able to reduce blood NfL in MS patients, suggesting that therapeutic remyelination acted on neuroprotection (Abdelhak et al., 2022). To test whether clemastine treatment is effective in patients with acute MS, another phase II double-blind placebo-controlled clinical trial (ReCOVER study; NCT02521311) is in the recruiting stage. Meanwhile, a phase II, randomized, double-blind, placebo-controlled trial of the ability of the combination of metformin and clemastine to promote remyelination in people with relapsing–remitting multiple sclerosis already on disease-modifying therapy (NCT05131828) is also at the recruiting stage. In addition, there is another clinical trial intended to assess clinical evidence of clemastine fumarate as a myelin repair therapy in patients with chronic inflammatory injury-causing demyelination as measured by multi-parametric MRI assessments (NCT05359653).

In mouse model studies, experimental autoimmune encephalomyelitis (EAE) and toxin-induced demyelination are the two major animal models used in MS research (Sutiwisesak et al., 2021). Expression of acetylcholine receptors has been reported in oligodendrocytes. M1 muscarinic acetylcholine receptor (CHRM1) is one of the main subtypes expressed in OPCs that negatively regulates cell differentiation and myelination (De Angelis et al., 2012). In the EAE mouse model, CHRM1 on OPCs was identified as the target of anti-muscarinic treatment for remyelination. Clemastine could accelerate remyelination, prevent axonal loss, and improve functional recovery specifically by targeting CHRM1 (Mei et al., 2016). In EAE rat model, clemastine showed protective role against neuroinflammation, oxidation, and demyelination by hindering p38 MAPK/NLRP3 signaling and Nrf2/HO-1/NLRP3/caspase-1/IL-1β pathway (Motawi et al., 2023). Glutathione S-transferase 4α (Gsta4) is highly expressed during adult oligodendrocytes differentiation. Clemastine activated Gsta4, and then restricted oligodendrocytes apoptosis and enhanced myelination, suggesting Gsta4 as a potential target of clemastine for MS therapies (Carlström et al., 2020). In lysolecithin-induced demyelination mice, exercise enhances oligodendrogenesis, remyelination. Moreover, exercise combined with clemastine additively enhance remyelination (Jensen et al., 2018), which provide evidence that combinations of clemastine with other different approaches may achieve a better therapeutic effect in MS.

*In vitro*, the neural progenitor cells from blood samples of MS patients were treated with or without clemastine for two consecutive days, and then the conditioned media was collected to culture primary rat OPCs. Clemastine in conditioned media enhanced OPCs differentiation and improved OPC maturation, further indicating the therapeutic potential of clemastine for patients with progressive MS (Nicaise et al., 2017).

### 2.2. Amyotrophic lateral sclerosis

Amyotrophic lateral sclerosis (ALS) is a progressive, paralytic neurodegenerative disease that characterized by degeneration of motor neurons and axonal degeneration in the brain and spinal cord (Feldman et al., 2022). The standardized global incidence of ALS is 1.68 per 100,000 persons each year (Marin et al., 2017). Cu/Zn SOD1 mutations are the most frequent types in ALS. Transgenic mouse model SOD1^G93A^ overexpresses the human protein with glycine for alanine substitution in position 93 and has been widely used for preclinical studies (Bonifacino et al., 2021). In SOD1^G93A^ mice, chronic clemastine administration reduced microgliosis, modulated inflammatory genes, and enhanced motor neuron survival. Moreover, *in vitro*, clemastine reduced activation of CD68-positive macrophages/microglia and inhibited pro-inflammatory reactions (Apolloni et al., 2016b). Interestingly, short treatment (from 40 days to 120 days, last for 80 days) and long treatment (from 40 days to the end of life, last for about 125 days) by clemastine in SOD1^G93A^ mouse showed opposite effects. Short treatment with clemastine could decrease inflammatory parameters and stimulate autophagic flux via the mTOR signaling pathway, thereby prolonging survival in SOD1^G93A^ mice. However, long treatment with clemastine failed to ameliorate ALS disease progression. Instead, long treatment with clemastine increased microgliosis and SOD1 protein levels in the late phases of the diseases. These differences in phenotypic outcomes may due to clemastine possesses complex and dual functions in the pathogenesis of ALS (Apolloni et al., 2016a). As the SOD1^G93A^ mouse model is just one of the genetic mouse models of ALS, therefore, further investigation is necessary to test the effects of clemastine on other ALS rodent models, such as TDP-43 mouse models, *FUS* mice, and *C9orf72* knock-out mice (Bonifacino et al., 2021), and to identify suitable dose that can protect against ALS damage.

### 2.3. Alzheimer’s disease

Alzheimer’s disease (AD) is the most common cause of dementia in the old. By 2050, the prevalence of dementia will triple worldwide based on a biological definition of AD (Scheltens et al., 2021). The memory impairments of AD correlate with the accumulation of amyloid-β plaques and Tau protein (Bloom, 2014; Puzzo et al., 2020). Myelin injury, neuroinflammation, synaptic and neuronal loss, and other neuropathological changes (Yang et al., 2017; Mathys et al., 2019; Leng and Edison, 2021; Pereira et al., 2021; Ashleigh et al., 2022; Blanchard et al., 2022) have also present in patients and animal models of AD. Animal studies suggest that clemastine has neuroprotective effects on AD-related memory deficits. In APP/PS1 transgenic mice, clemastine could decrease amyloid-β generation and accumulation by reducing beta-site amyloid precursor protein cleaving enzyme 1 (BACE1), which requires for the monomeric forms of amyloid-β. In addition, clemastine mitigated neuroinflammation and enhanced autophagy by suppressing mTOR signaling, ultimately attenuating AD-like pathology (Li et al., 2021). Clemastine also prevented OPCs from entering the state of cellular senescence, which facilitating myelin formation in AD mice (Xie et al., 2021). It is worthy to mention that another research showed that clemastine did not change the number of amyloid-β plaques in different brain regions of APP/PS1 brains, implying that clemastine rescued cognitive decline in AD mice mainly through enhancing myelin renewal instead of altering amyloid-β deposition or clearance (Chen J. F. et al., 2021). Currently, the pharmacological mechanisms of clemastine in AD are limited, further researches are required to fully understand the potential therapeutic targets of clemastine in this disease.

### 2.4. Perioperative neurocognitive disorders

With the worldwide increase in lifespan, surgical patients are becoming older. Perioperative neurocognitive disorder (PND) is the most common complication experienced by older individuals undergoing anesthesia and surgery. PND is an umbrella term including preexisting cognitive impairment, preoperative delirium, delirium occurring up to 7 days after surgery, delayed neurocognitive recovery, and postoperative neurocognitive disorders that last for 30 days and 12 months after surgical procedures (Evered et al., 2018). Next-generation sequencing and bioinformatics predicted clemastine had therapeutic potential on PND (Wu et al., 2021a). Clemastine was given at 10 mg/kg per day for 2 weeks to evaluate its effects on PND in aged mice. As expected, clemastine blocked the overactivation of the Wnt/β-catenin signaling pathway, further enhancing OLs differentiation and remyelination. Additionally, clemastine also reduced neuroinflammation, improved synaptic plasticity, and prevented the loss of hippocampal mature neurons (Wu et al., 2021b). The forementioned findings provide some insight into the positive effects of clemastine in PND.

## 3. Experimental studies of clemastine in environmental and genetic induced brain development disorders

Environment and genetics are major contributors to affect neurodevelopment in the early life (Champagne, 2010). Throughout early neurodevelopment, myelination helps provide the foundation for brain connectivity and supports the maturation of cognitive functioning. If myelination progress is affected by nutritional deficiencies, environmental neurotoxins, or genetic abnormality, this may lead to permanent alterations in brain function, and have detrimental consequences for long-term behavior (Li et al., 2019; McCann and Soriano, 2019; Raper et al., 2021). Clemastine can alleviate environmental and genetic induced brain development disorders by promoting myelination.

### 3.1. Omega-3 fatty acids (n-3 PUFAs) deficiency related disorder

Lipids are one of the main constituents of the CNS. Docosahexaenoic acid (DHA, omega-3) is one of the principal form of the long chain polyunsaturated fatty acid of the white matter (Martinez, 1992). Low maternal intake of n-3 PUFAs leads to neurodevelopmental disorders and defects in brain functional connectivity (Madore et al., 2020). Recent data suggested that decreasing n-3 PUFA dietary intake led to deficits in postnatal oligodendrocytes maturation and myelination processes in mice. N-3 PUFA defiieny then resulted in cognitive and emotional disorders in adult life. Clemastine could enhanceoligodendrocyte precursor cell differentiation, promote myelination, and rescue memory function in n-3 PUFA deficiency mice (Leyrolle et al., 2022). Since n-3 PUFAs are lipids possess anti-inflammatory function, n-3 PUFAs deficiency may lead to pro-inflammatory condition in CNS, Madore et al. (2020). It is plausible that clemastine promoted OLs maturation by modulating brain inflammation.

### 3.2. Anesthetics induced development disorder

Isoflurane and sevoflurane are commonly used anesthetics. Studies showed that isoflurane induced apoptosis of OLs and impaired neural function in neonatal primate and rodent brains (Brambrink et al., 2012; Creeley et al., 2014; Li et al., 2019). After exposure to isoflurane, mice were fed with clemastine (10 mg/kg/day) from postnatal days 21 to 35, the critical period of myelin development. Data revealed that clemastine enhanced myelination and could reverse cognitive dysfunction induced by isoflurane (Li et al., 2019). Similarly, clemastine injected intraperitoneally (10 mg/kg/day) in mice on postnatal days 6 to 8 enhanced OLs maturation and myelination, and thus alleviated sevoflurane-induced cognitive impairment (Zhang et al., 2022), indicating that although doses differed, long-term and short-term treatments with clemastine could rescue developmental cognitive impairment induced by anesthetics.

### 3.3. Williams syndrome

Williams syndrome, caused by a heterozygous microdeletion on chromosome 7q11.23, is a multisystemic neurodevelopmental disorder characterized by hypersociability with unique cognitive and personality profiles (Kozel et al., 2021). General transcription factor IIi (Gtf2i) is located on adjacent loci at the telomeric end of the Williams syndrome critical region, which linked to typical WS behavior and development. In Gtf2i cKO mice, clemastine treatment normalized the number of oligodendrocytes in the cortex and the corpus callosum, and increased myelin thickness. These results in rescuing the myelination deficit further normalize the behavioral deficits (Barak et al., 2019). These data imply that targeting myelination deficits by clemastine might be a beneficial therapeutic strategy in Williams syndrome.

### 3.4. Autism spectrum disorder

Pitt-Hopkins syndrome, a syndromic form of autism spectrum disorder (ASD), is a neurodevelopmental disorder characterized by intellectual disability, specific facial features, deficits in motor learning, and marked autonomic nervous system dysfunction (Phan et al., 2020). Pitt-Hopkins syndrome is caused by haploinsufficiency of the transcription factor 4 gene (TCF4) on chromosome 18q21. Recent study found that clemastine is benificial at restoring myelination in a Pitt-Hopkins syndrome mouse model. Clemastine treatment normalized OPCs and oligodendrocyte density *in vivo* and *in vitro*. Importantly, clemastine helped functional recovery by improving electrophysiology and behavior. The preclinical evidence indicated that clemastine may be beneficial in Pitt-Hopkins syndrome (Bohlen et al., 2023).

However, a recent study found clemastine-induced impairment in developmental myelination in healthy mice (Palma et al., 2022). C57BL/6 wild-type mice were treated with clemastine (50 mg/kg/day) by intraperitoneal injections from postnatal day 5 to postnatal day 21. This literature found that clemastine resulted in the decreasement of conduction velocity of myelinated fibers in mice, despite the increase of oligodendrocyte differentiation. These data implied that clemastine’s impact on neurodevelopment brain is complex. It seems like that clemastine protects the developing brain from damage, but has little effect or is even detrimental to normal neural development.

## 4. Effects of clemastine in experimental studies of brain injury

Preclinical and clinical evidence illustrates the beneficial effects of clemastine in brain injury, especially in hypoxic brain injury, hypoxic–ischemic brain injury, and spinal cord injury.

### 4.1. Hypoxic brain injury

Hypoxia, or insufficient supply of oxygen (O_2_) with respect to the demand, afflicts millions of people worldwide. Chronic hypoxia impairs myelination and synaptogenesis in developing brains, which leading to cognitive deficits in adolescents (Wang et al., 2018; Koehn et al., 2020).

Clinical treatments supported the therapeutical effect of clemastine on hypoxia-related cognitive dysfunction. White matter injury and demyelination following necrosis of vulnerable oligodendrocytes is one of the pathophysiology of delayed post-hypoxic leukoencephalopathy, a unique clinical condition that presents with cognitive impairment occurring following an episode of acute hypoxic brain injury (Hamlin et al., 2020). A 45-year-old male patient who developed delayed post hypoxic leukoencephalopathy was given clemastine, 5.36 mg, twice daily for 10 months. After the treatment, magnetic resonance imaging (MRI) showed an improvement and nearly complete resolution of the diffuse white matter hyperintensity, and the cognitive function of this patient had markedly improved as well (Cree et al., 2018).

In neonatal hypoxic injury mice model, clemastine promoted OPC differentiation, myelination, and improved cognitive functional recovery. The action of clemastine in hypoxia was oligodendroglial specific via the CHRM1 (Cree et al., 2018). Consistent with this study, another study also found that clemastine administration during or after hypoxia exposure could enhance oligodendroglial differentiation and facilitate functional recovery in neonatal hypoxic injury mice (Wang et al., 2018). Not only that, clemastine also protected adult mice brain functions from hypoxia. 4-month-old mice suffered from hypoxia were given clemastine for 4 weeks. As a result, clemastine increased the newly-formed myelin in the motor cortex and corpus callosum (Chen L. et al., 2021). As mentioned, glial cell interactions regulate myelination processes and affect myelin functions. In addition to improving myelin repairment directly, clemastine mediated its therapeutic effect by inhibiting the microglia-induced inflammatory response (Zhi et al., 2021). In hypoxic–ischemic brain injury, IL-1β was released by activated microglia, which resulting in hypomyelination. Further, co-culture of microglia and OPCs cell experiment illustrated that clemastine could inhibit the production of IL-1β by inhibition of the p38 MAPK/NLRP3 pathway in microglia cells, and then promote the maturation of OPCs. These data indicated that clemastine might be a viable strategy to promote myelination in hypoxic–ischemic brain injury models (Xie et al., 2020).

### 4.2. Spinal cord injury

Spinal cord injury (SCI) is a traumatic injury that affects the normal function of the spinal cord (Anderson et al., 2022). Loss of OLs and demyelination of spared axons lead to slower or blocked conduction through the lesion site caused by SCI. In SCI rat model, clemastine preserved myelin integrity, and thus improved functional recovery (Du et al., 2022). One mechanism was that clemastine enhanced OPCs differentiation and myelin wrapping (Tong et al., 2022). Clemastine could activate ERK1/2 via CHRM1 in OPCs, and promote cell differentiation (Tong et al., 2022). Clemastine also negated cell death and improved remyelination and recovery of cognitive function (Myatich et al., 2023). Thus clemastine potentially serves as an efficient drug treatment in the recovery from SCI.

## 5. Experimental studies of clemastine in psychiatric disorders

Mylin damage and neuroinflammation occurs in psychiatric disorders. Recently evidence has indicated that clemastine also presented therapeutic potential in animal models of major depressive disorder and schizophrenia.

### 5.1. Major depressive disorder

Depressive disorder is one of the most prevalent and debilitating psychiatric disorders. In 2019, depressive disorders were the second leading cause of disability worldwide (GBD 2019 Mental Disorders Collaborators, 2022). Characterized by impairments in cognition, emotional regulation, and neurovegetative symptoms, the major depressive disorder can cause severe disability (Park and Zarate, 2019). The pathophysiology of depressive disorders includes increased inflammation, decreased neurogenesis and neuroplasticity, and abnormal myelination (Fries et al., 2022). Several studies have shown that clemastine ameliorated depressive-like behaviors by targeting different pathological changes. On the one hand, clemastine enhanced the process of myelination. Epigenetic modification of repressive histone methylation is critical for gene repression during oligodendrocyte differentiation (Liu et al., 2015). Clemastine could induce OPCs differentiation by enhancing the activity of H3K9 histone methyltransferases and favoring chromatin compaction, thereby reversing depressive-like social behavior (Liu et al., 2016). It has also been observed that clemastine rescued the behavioral abnormalities accompanied by increasing oligodendrogenesis in stress model of social defeat (Shimizu et al., 2020). On the other hand, clemastine alleviated depressive-like behavior by inhibiting microglia-related pro-inflammatory response (Su et al., 2018). It is well-known that purinergic ligand-gated ion channel 7 receptor (P2X7R) is present on activated microglia (Bhattacharya and Biber, 2016). Evidence suggested that clemastine suppressed the expression of P2X7R and restrained the microglia activation (Su et al., 2018).

### 5.2. Schizophrenia

The systematic analysis for the Global Burden of Disease Study reported that the prevalence of schizophrenia was 23.6 per million worldwide in 2019 (GBD 2019 Mental Disorders Collaborators, 2022). Clinical observation found that schizophrenia patients exhibited white matter volume consistent with microstructural disorganization and axonal dysfunction (Park et al., 2014; Caprihan et al., 2015). To date, few or no effective drug is available for the myelination related deficits that may underly cognitive deficits in schizophrenia. Animal study revealed that targeting oligodendrocytes and myelin repair might be beneficial to cure schizophrenia like behaviors (Yu et al., 2022). Mice exposed to cuprizone displayed schizophrenia-like behavioral changes, clemastine enhanced myelin repairment in demyelinated regions of the brain and rescued memory and anxiety related behavioral deficits (Li et al., 2015). However, other cognitive domains need to be investigated in more schizophrenia-related animal models.

## 6. Conclusion and future perspectives

**Figure 2:**
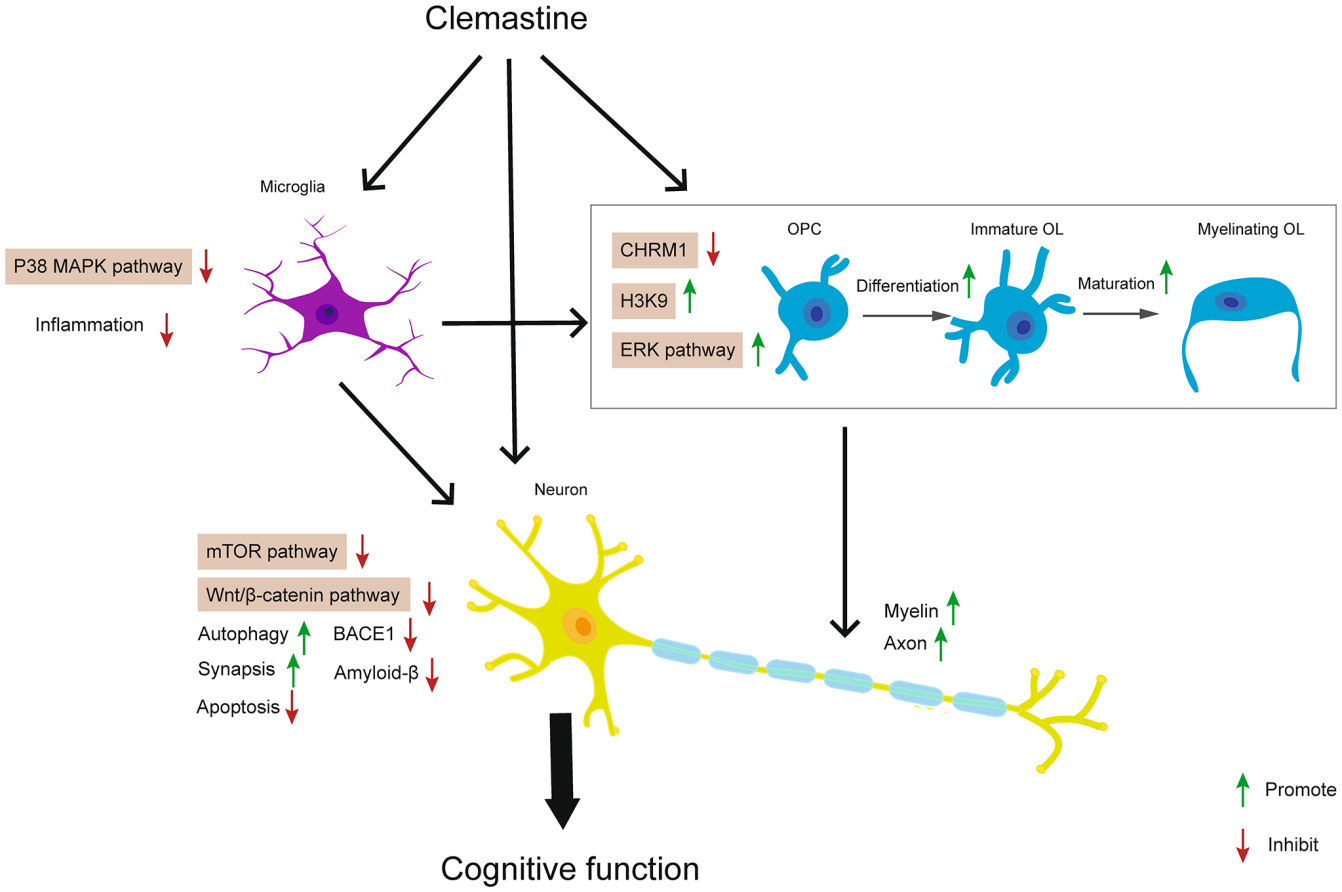
Effects of clemastine on neurological disorders and potential signaling pathways.

In conclusion, we summarized current evidence about the neuroprotective effects of clemastine in neurodegenerative diseases, neurodevelopmental deficits, brain injury, and psychiatric disorders. In these processes, clemastine regulates CHRM1, p38 MAPK, ERK, mTOR, and Wnt/β-catenin signaling pathways in OPCs, OLs, microglia, and neurons. The underlying mechanisms of action of clemastine are generally associated with promoting oligodendrocytes differentiation and maturation, inhibiting microglia induced neuroinflammation, and decreasing the apoptosis of neurons (Figure 2). Therefore clemastine might be a promising candidate compound and repurposing for various neurological disorders.

Nevertheless, only a few clinical trials assessed the efficacy and safety of clemastine as a therapy for patients with MS. Until now, there is still lacking confirmed clinical trials about the amelioration of clemastine in patients with other CNS disorders. Besides, clemastine shows remyelination and functional repair over short timescales in phase II clinical trials. It needs to explore whether clemastine still has beneficial effects beyond clinical study. Furthermore, the dose of clemastine selected in the clinical trials only achieves partial saturation of the target muscarinic receptor. Future investigations aims at the optimal dosage may hold promise as potential therapeutic strategies for clinical effect of treatment (Green et al., 2017). Experiments have implied that clemastine may cause immune suppression (Peternel et al., 2006; Johansen et al., 2011). C57BL/6 mice have a high resistance to *listeria monocytogenes,* but clemastine enhanced the susceptibility of mice to *listeria monocytogenes* infection, augmented listerial caused liver abscesses and disruption of splenic architecture, leading to death. In human monocytes, clemastine inhibited the potential of cells to produce antibacterial cytokines in response to *listeria* or lipopolysaccharide (Johansen et al., 2011). Besides clemastine has antihistamine property with anticholinergic and sedative side effects. Generally, neurological disorders require long-term therapy, thus whether clemastine long treatment causes immune suppression or other pathological change, like its effects in ALS, are not clear. More clinical trials with long-term follow-ups are needed to validate the efficacy of clemastine for CNS diseases. Additionally, the mechanisms underlying the positive impact of clemastine during neuropathological disorders remain unclear. However, preclinical studies mainly focused on one signaling pathway of clemastine in specific cell types. Therefore, further investigations that aimed at unraveling the detailed molecular mechanisms and identifying therapeutic targets for CNS damage are necessary.

## Author contributions

SJ: Writing – review & editing, Writing – original draft. XW: Writing – original draft, Writing – review & editing. TC: Writing – review & editing. RK: Writing – review & editing, Supervision, Visualization. LH: Supervision, Visualization, Writing – review & editing, Funding acquisition, Project administration.

## Funding

The author(s) declare financial support was received for the research, authorship, and/or publication of this article. This work was supported by the National Natural Science Foundation of China (82071901).

## Conflict of interest

The authors declare that the research was conducted in the absence of any commercial or financial relationships that could be construed as a potential conflict of interest.

## Publisher’s note

All claims expressed in this article are solely those of the authors and do not necessarily represent those of their affiliated organizations, or those of the publisher, the editors and the reviewers. Any product that may be evaluated in this article, or claim that may be made by its manufacturer, is not guaranteed or endorsed by the publisher.
